# What Mothers Can Do

**Published:** 1861-06

**Authors:** 


### WHAT MOTHERS CAN DO.

FORTY-TWO years ago there was born to the wife of a poor and obscure blacksmith, a son. The father died, and, soon after, the mother; and their history and memory perished from before men. The infant child was left to the care of whomsoever might take a fancy to it; but as months passed, then years, one friend took it up and then another; and how, he could scarcely tell himself, he obtained a collegiate education and found his way into the ministry; when, one day, a thousand miles away from the play-grounds of his childhood, after preaching to a large attentive audience, an old lady met him at the foot of the pulpit-stairs and said: "I was present at your birth: I knew your mother well, and I do not wonder you have risen to be a minister of the Gospel, for it was her habit to give you to the Lord in prayer before you were born." Blessed mother! unknown to the rich and great of her time, known, perhaps, even to her neighbors only as the "blacksmith's wife," she worked, and lived, and loved, and prayed in her poor little obscure sphere, until it was her Master's will that she should go up higher; and she went early, because she was early ready; but her works follow after and upward unto heaven, as one by one souls saved by her son's instrumentality cross over Jordan, and meeting her with other angels bright on the better bank, they join hand to hand and file away upward to the Father's bosom, chanting in glory: "Saved by grace through her prayers."

More than a hundred years ago there lived in London the wife of a sea-captain: who were her ancestors, where she was born, or what of her life, no one knows or ever will know now. She was early left a widow with a fatherless child; but she feared God and felt her responsibilities to the child of her love. But in spite of a mother's teachings he went to sea and became one of the most profligate of young men; but never, in all his wanderings and dissipations, could he rid himself of the remembrance of the sad, pale, and sweet face of his mother, nor her earnest, patient, and loving teachings. She died, but her prayers bound him fast to the throne of God, and John Newton became one of the best of men. His pious conversation was the means of converting Dr. Buchanan, whose work, *Star in the East*, led Adoniram Judson to the Saviour, converted Dr. Scott, the commentator; Cowper's piety was deepened, Wilberforce became a changed man, and wrote a Practical View of Christianity, which converted Legh Richmond, who wrote the *Dairyman's Daughter*, and how many souls that book has awakened and led to the Saviour, and will continue to do, only the records of eternity can tell. Mothers! however poor, and obscure, and unknown, look upon your boy-child and remembering what God hath wrought through such as you, take courage, and pray in faith that the same he can do by you.

